# Comment on Sun et al. First Experience with Hypothermic Oxygenated Perfusion in Human Uteri: Feasibility and Metabolic Characterization. *J. Clin. Med.* 2026, *15*, 2820

**DOI:** 10.3390/jcm15124695

**Published:** 2026-06-17

**Authors:** Ludivine Dion, Carla Sousa, Margot Dugast, Vincent Lavoué

**Affiliations:** Inserm, UMRS 1085, IRSET (Institut de Recherche en Santé, Environnement et Travail), CHU Rennes, University of Rennes, F-35000 Rennes, Francevincent.lavoue@chu-rennes.fr (V.L.)

We read with great interest the recent article by Sun et al. describing the first application of hypothermic oxygenated perfusion (HOPE) in human uteri, titled “First Experience with Hypothermic Oxygenated Perfusion in Human Uteri: Feasibility and Metabolic Characterization” by Keyue Sun et al [1], published on 8 April 2026. This innovative work represents a major step forward in improving graft preservation and viability assessment in uterus transplantation (UTx), particularly in the context of deceased donors, where prolonged ischemia remains a critical limitation; around 6 h in the case of a deceased donor, compared with 2.3 h in the case of a living donor [2].

As emphasized by the authors, ischemia/reperfusion injury is a major determinant of graft outcome in Utx [3]. Machine perfusion strategies such as HOPE have already demonstrated significant benefits in other solid organs, particularly through mitochondrial protection and metabolic recovery. In this context, the translation of these approaches to uterine grafts is both timely and highly relevant.

However, we would like to highlight an important technical consideration that may significantly impact perfusion quality and graft evaluation: arterial perfusion modalities.

In the study by Sun et al., both uterine arteries were perfused, using a single pump for both cannulas, connected via a Y-connector. While this configuration allows for technical feasibility, it may not adequately reflect the physiological vascularization of the uterus. Indeed, the uterus is supplied by two uterine arteries that may exhibit different vascular resistances and flow dynamics. Consequently, a single-pump system may result in uneven perfusion distribution between the two sides of the uterus.

Based on our previous experimental work on uterine machine perfusion models, we believe that bilateral perfusion using two independent pumps offers several key advantages [4,5].

First, independent perfusion enables precise control of pressure and flow in each uterine artery, thereby ensuring a more homogeneous organ perfusion. This is particularly important given the potential asymmetry in vascular resistance between the two arterial systems.

Second, thrombosis remains the leading cause of graft failure in uterus transplantation. During procurement and preservation, thrombi may form and lead to regional perfusion defects. In a single-pump system, such asymmetries may remain undetected. In contrast, a dual-pump configuration allows for real-time monitoring of arterial resistance on each side, thereby facilitating the early identification of vascular obstruction.

Third, this approach is supported by the existing literature. A recent comprehensive review reported that most human studies using machine perfusion in uterus transplantation rely on bilateral perfusion with separate pumps, highlighting the relevance of this strategy for both experimental and clinical applications [6].

Taken together, these considerations suggest that single-pump perfusion using a Y-connector may be suboptimal when the objective is not only preservation but also accurate graft assessment. Bilateral and independent perfusion may improve both perfusion homogeneity and the detection of vascular abnormalities. We have summarized the comparison between these two concepts in Figure 1.

In conclusion, we commend the authors for their pioneering work, which represents an important milestone in the field of uterus transplantation. Further optimization of perfusion strategies, particularly regarding arterial inflow configuration, may help maximize the clinical impact of machine perfusion and contribute to expanding the use of deceased donor uteri.

## Figures and Tables

**Figure 1:**
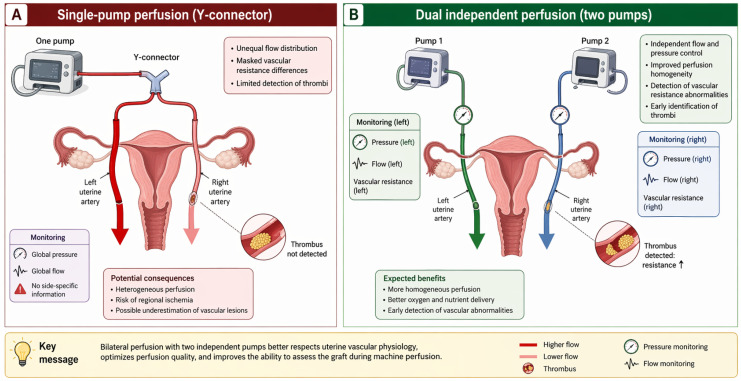
Using a single-pump perfusion system (**A**), flow distribution may be unequal between the two sides of the uterus, potentially resulting in regional ischemia within the graft or an underestimation of vascular lesions. In contrast, using two pumps (**B**) enables independent perfusion through each uterine artery, allowing for a more homogeneous flow distribution. This approach is expected to improve oxygen delivery and facilitate the early detection of vascular abnormalities.
